# Improving the Efficiency of Bulk-heterojunction Solar Cells through Plasmonic Enhancement within a Silver Nanoparticle-Loaded Optical Spacer Layer

**DOI:** 10.1021/acsomega.4c08801

**Published:** 2025-01-13

**Authors:** Mohammed A. Ibrahem, Bassam G. Rasheed, Betul Canimkurbey, Ali M. Adawi, Jean-Sebastien G. Bouillard, Mary O’Neill

**Affiliations:** 1 Laser Sciences and Technology Branch, Applied Sciences Department, University of Technology, Baghdad 10066, Iraq; 2 Laser and Optoelectronics Engineering Department, College of Engineering, Al Nahrain University, Baghdad 10066, Iraq; 3 UNAM − Institute of Materials Science and Nanotechnology and National Nanotechnology Research Center, Bilkent University, Ankara 06800, Turkey; 4 Serefeddin Health Services Vocational School, Central Research Laboratory, Amasya University, Amasya 05100, Turkey; 5 Department of Physics and Mathematics, University of Hull, Kingston upon Hull HU67RX, United Kingdom; 6 School of Science and Technology, Nottingham Trent University, Clifton Lane, Nottingham NG11 8NS, United Kingdom

## Abstract

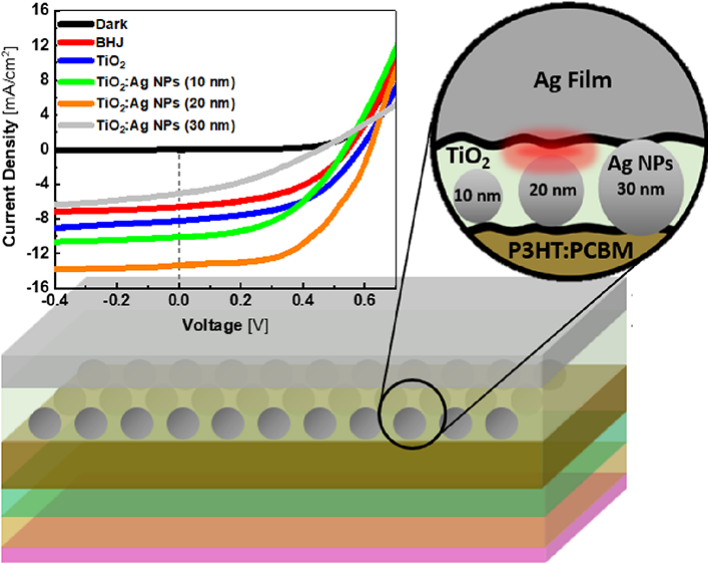

We investigate the enhancement in the efficiency of organic bulk heterojunction solar cells enabled by plasmonic excitation of Ag nanoparticles (NPs) of different diameters (10, 20, and 30 nm), randomly incorporated within an optical spacing layer of TiO_2_ placed between the organic medium and the Ag cathode. Such structures significantly increase the optical absorption and the photocurrent within the device system, leading to a power conversion efficiency of more than 4%, over 2.5 times that of the control bulk heterojunction cell. This corresponds to a 61% increase in *J*_*SC*_ and a 6.3% in fill factor. 3D Finite-difference time-domain simulations were utilized to investigate the plasmonic field coupling within the nanogap medium of TiO_2_. They show that coupling between the Ag nanoparticle and the Ag thin film cathode extends the wavelength range of the local field enhancement beyond that obtained for isolated NPs, providing a better overlap with the absorption spectrum of the organic medium.

## Introduction

Organic photovoltaics (OPVs) have been the subject of intense investigation in the last two decades due to their promising future as a renewable and sustainable energy production source.^1−5^ Despite their low cost, lightweight, and ease of process on flexible surfaces, organic photovoltaics have inefficient charge separation and collection since the carrier diffusion length is usually significantly less than the optical absorption length (∼100 nm).^6,7^ Bulk heterojunction (BHJ) solar cells, enabled by mixing the electrons donor and acceptor solvents, partially solve this fundamental problem.^8^ However, optimized spin-coated laboratory-scale BHJ devices are still too thin to convert all the incoming light into photocurrent.^7,9,10^ To overcome these limitations, maximizing light interactions inside the solar cell device is a promising approach to enhancing the device’s power conversion efficiency.^11−14^

Heeger et al.^15^ proposed a new device architecture based on redistributing the internal electric field inside the organic solar cell to maximize light absorption over the active medium. Such absorption enhancement is achieved by inserting a thin optical spacing layer of TiO_*x*_ with a 30 nm thickness between the active medium and the back electrode. As a result, the device’s power conversion efficiency is improved by over 50%.^16,17^ Another promising solution is to harness the plasmonic effect of noble metal nanostructures with spectral resonances within the absorption spectrum of the solar cell.^18−20^ These metal nanostructures can efficiently absorb/scatter light at specific frequencies and act as a subwavelength nanoantenna, significantly enhancing the local electric field within a few nanometers spacing around them.^21−24^ Metal nanoparticles also have a wide range of applications, such as light-harvesting centers, novel electronic devices, and nanoprobes in medical devices.^25−100^ Plasmonic nanoparticle-induced power conversion efficiency enhancement is well-reported in organic photovoltaics.^4,27−29^ There is extensive literature on strategies for integrating different plasmonic nanostructures within distinct regions of the device structure to improve its performance.^30−35^ For instance, Baek et al.^36^ improved the device performance by incorporating Ag NPs of 67 nm inside the hole transporting layer of poly(3, 4-ethylenedioxythiophene): poly(styrenesulfonate) (PEDOT:PSS) layer. Ng et al.^37^ improve solar cell absorption by introducing Au nanostructures of different sizes and shapes within the PEDOT:PSS layer. In a recent article by Nair et al.,^38^ device power conversion efficiency was enhanced by 15% when Ag NPs were incorporated within the cathode buffer layer and the active medium. Karakurt et al.^39^ showed a power conversion efficiency increase of 21.4% by adding Au NPs to the device’s active medium. A recent review by Liu et al.^21^ summarizes effective strategies for adding plasmonic nanostructures in different locations within the organic solar cells for efficiency enhancement. Plasmonic nanocavity configurations have been explored to enhance the efficiency of photovoltaic devices through the excitation of waveguide modes.^40−42^ Simulations predicted that the excitation of waveguide modes to enhance the performance of photovoltaic devices using metallic nanoparticles is feasible.^43^

Ag NPs have been widely used in organic solar cells due to their chemical stability, low cost, and ease of synthesis compared to other plasmonic metal nanoparticles.^44^ Their utilization in solar cell devices is based on their immense capability of absorbing and trapping incident light through localized plasmon resonances, enabling high photocurrent generation and improving the overall device efficiency. This work investigates the plasmonic coupling between the Ag nanoparticles and the Ag back electrode to form a plasmonic nanogap inside the device structure. Plasmonic nanogaps have been shown to enhance light-matter interactions,^45−51^ and we characterize the impact of their inclusion on the overall device performance, which will have significant practical implications for developing cost-effective and efficient solar cell technologies. The novelty in our work is that we address and examine the possible plasmonic coupling between the incorporated Ag nanoparticles and the Ag back electrode to form a plasmonic nanogap, which has been shown to enhance light-matter interactions and the impact on the overall device performance.

Herein, we demonstrate a 3-fold efficiency enhancement of BHJ solar cells by combining the effects of the optical spacing layer and the plasmonic resonance excitations. We investigate the efficiency of BHJ solar cells of (ITO/PEDOT/P3HT:PCBM/Ag) and (ITO/PEDOT/P3HT: PCBM/TiO_2_:Ag NPs/Ag) as a function of three different Ag NPs sizes (10, 20, and 30 nm) randomly distributed within a thin TiO_2_ layer. The TiO_2_ film is an electron-transporting layer as well as an efficient optical spacer layer that spatially redistributes the electrical field toward the active medium. We also theoretically study the spatial distribution of the plasmonic field stimulated by 20 nm Ag NPs, fully embedded in a TiO_2_ film of 30 nm thickness, and we investigate the coupling of the plasmonic field of the NP with an extended Ag film (the solar cell back electrode) using Lumerical FDTD software. These simulations suggest that coupling between the NPs and Ag film shifts the plasmonic resonances to longer wavelengths, providing better overlap with the absorption spectrum of the organic photoactive layer and enhancing the efficiency of the organic solar cell. This work introduces a new approach to effectively trap incident light within the device structure, thereby enhancing its overall performance. The simplicity of this approach may lead to a reduction in the production cost of organic solar cell devices involving plasmonic nanoparticles, rendering plasmonic solar cell devices economically viable.

## Experimental Method

Bulk heterojunction solar cell devices were prepared under optimized conditions using the following preparation procedure. The substrate was prepatterned 100 nm thick indium tin oxide (ITO) coated glass, with a sheet resistance of 20 Ω/sq and 1.8 nm RMS surface roughness purchased from Ossila. The substrates were cleaned thoroughly by sonication for 10 min with three consecutive solutions: DI water, acetone, and isopropanol, and then dried with compressed air. Poly(3,4-ethylenedioxythiophene)-poly(styrenesulfonate) (PDOT:PSS), purchased from Sigma-Aldrich, used as a hole transporting layer, is spin-coated on top of the ITO electrodes at 4000 rpm for 30 s then annealed in air at 150 °C for 10 min. The thickness of the PEDOT: PSS layer was approximately 40 nm. The BHJ active layer of P3HT: PCBM dissolved in chlorobenzene was spin-cast from 2.5% by weight, with the ratio of (1:0.6) from the donor and acceptor compounds, at 2000 rpm for 15 s in the glovebox filled with nitrogen. This was followed by thermal annealing of the active layer at 150 °C for 15 min. The P3HT: PCBM mixed solution is filtered with 0.45 μm PTFE filters before the deposition to remove any possible agglomeration or undissolved molecules. All fabricated devices have an active layer thickness of 120 nm (±3 nm).

TiO_2_, suspended in DI water at 0.5% w/v concentration, was purchased from Sigma-Aldrich and spin-coated on top of the BHJ active layer at 2000 rpm for 30 s, followed by annealing on the hot plate at 100 °C for 15 min in a nitrogen filled glovebox. The thickness of the optical spacing layer of TiO_2_ is 30 nm (±3 nm), as measured by a Tencor P-10 Profilometer. To investigate the plasmonic excitation effect on solar cell performance, the optical spacing layer of TiO_2_ is separately modified with Ag NPs of different sizes. The randomly distributed Ag NPs (10, 20, and 30 nm in diameter purchased from nanoComposix) in TiO_2_ films were prepared by mixing a diluted solution of 0.05 mg/mL water-suspended Ag NPs with 0.5% TiO_2_/DI water in 1:1 mixing ratio by volume, for each Ag particle size. The number density of the incorporated Ag NPs for each size is estimated to be 2 × 10^13^, 2.4 × 10^12^, and 7.7 × 10^11^ particles/ml for 10, 20, and 30 nm, respectively. The mixture was then sonicated, spin-cast, and annealed as described above. This process was followed by depositing 100 nm of Ag film by thermal evaporation at 2 × 10^–6^ Torr to form the back electrode of the solar cell. The solar cell devices were encapsulated with a thin glass coverslip before removing from the nitrogen environment.

Each device’s dark and photogenerated currents, having an overall area of 4.5 mm^2^, were measured using a Keithley 2400 source meter. The devices were illuminated with the solar simulator (Newport 94023A) at incident power (*P*_*in*_) of 100 mW/cm^2^ (1.5 AM). The solar cell power conversion efficiency (PCE) is calculated based on the following equation:^52,53^
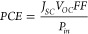
1where *J*_*SC*_, *V*_*OC*_ and *FF* are the short circuit current density, open circuit voltage, and fill factor, respectively. The external quantum efficiency (*EQE*) of the devices is also measured under short circuit conditions based on the following equation:^54^
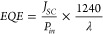
2where λ is the incident wavelength. Measurements were carried out on 2 devices for each device geometry, giving a total number of 10 devices.

## Results and Discussion

**Figure 1:**
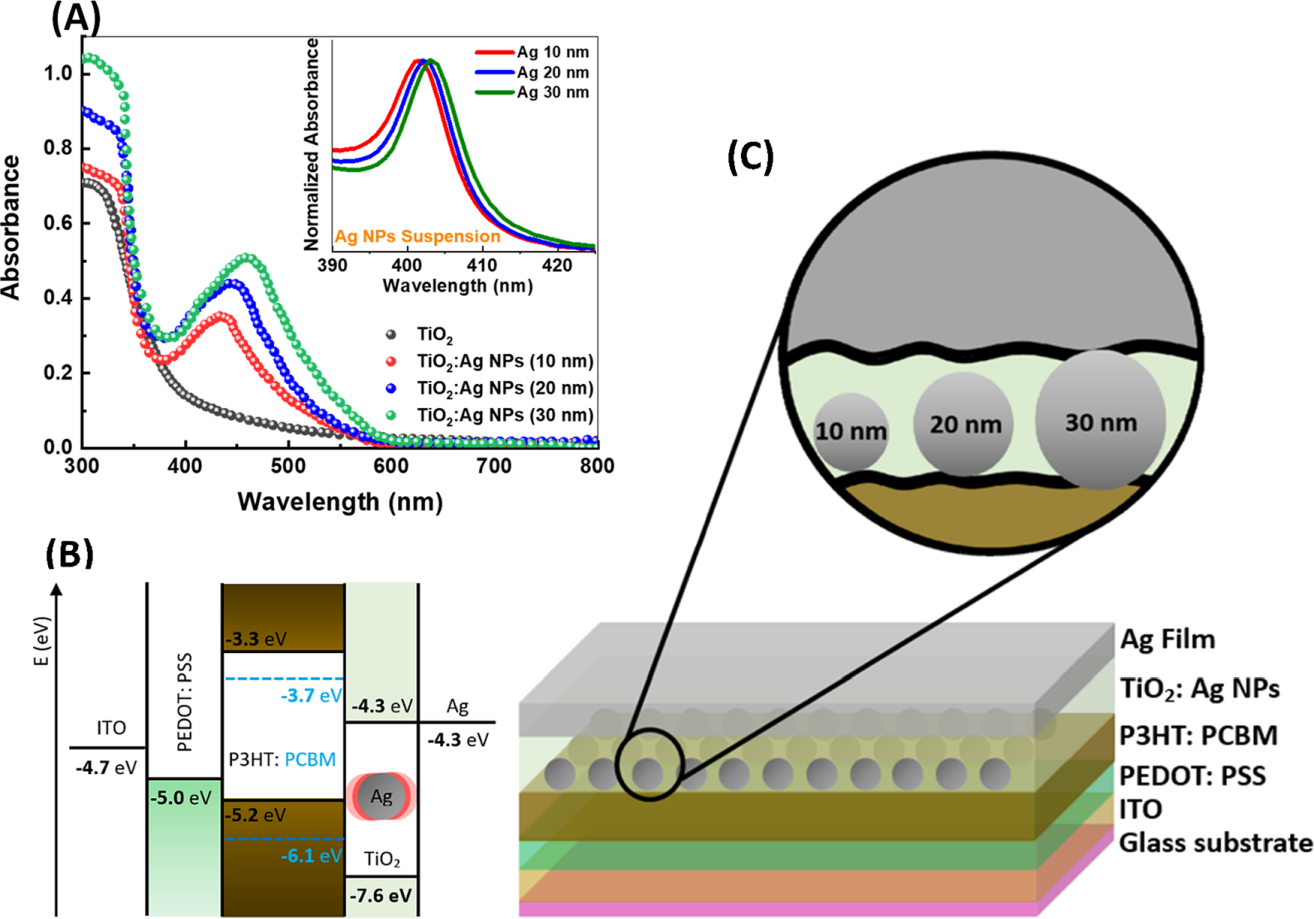
(A) Optical absorption of TiO_2_ film with and without incorporating Ag NPs with different particle sizes (10, 20, and 30 nm). The inset shows the optical absorption of Ag NPs of different sizes in suspension. The red shift in the absorption peak with increasing size is attributed to the localized surface plasmon effect of Ag NPs inside the TiO_2_ film. (B) Energy level diagram of the device structure utilized in this work. The diagram shows the published values of the valence band, conduction band, and the Fermi level of the device structure.^15,35,55,56^ (C) Device configuration illustrating the incorporation of TiO_2_ optical spacing layer containing Ag NPs with different particle sizes.

Figure 1A shows the absorption spectra of the optical spacer layer of TiO_2_ with and without Ag NPs of different diameters. The inset of Figure 1A shows the plasmonic resonance absorption of Ag NPs in suspension, where a slight redshift is observed when the Ag NP diameter is increased. The thin film spectra show the signature absorption peak of TiO_2_ in the UV due to the interband electron transitions and the nanoparticle’s plasmonic resonance peaks at 434, 445, and 460 nm for the Ag NPs of diameters 10, 20, and 30 nm, respectively. The absorption intensity also significantly increases with Ag NP size due to the light scattering effect. Figure 1B illustrates the energy level alignment of the various layers across the fabricated BHJ solar cell device. Figure 1C depicts the BHJ solar cell structure, showing Ag NPs blended in the TiO_2_ optical spacer layer with a thickness of 30 nm.

**Figure 2:**
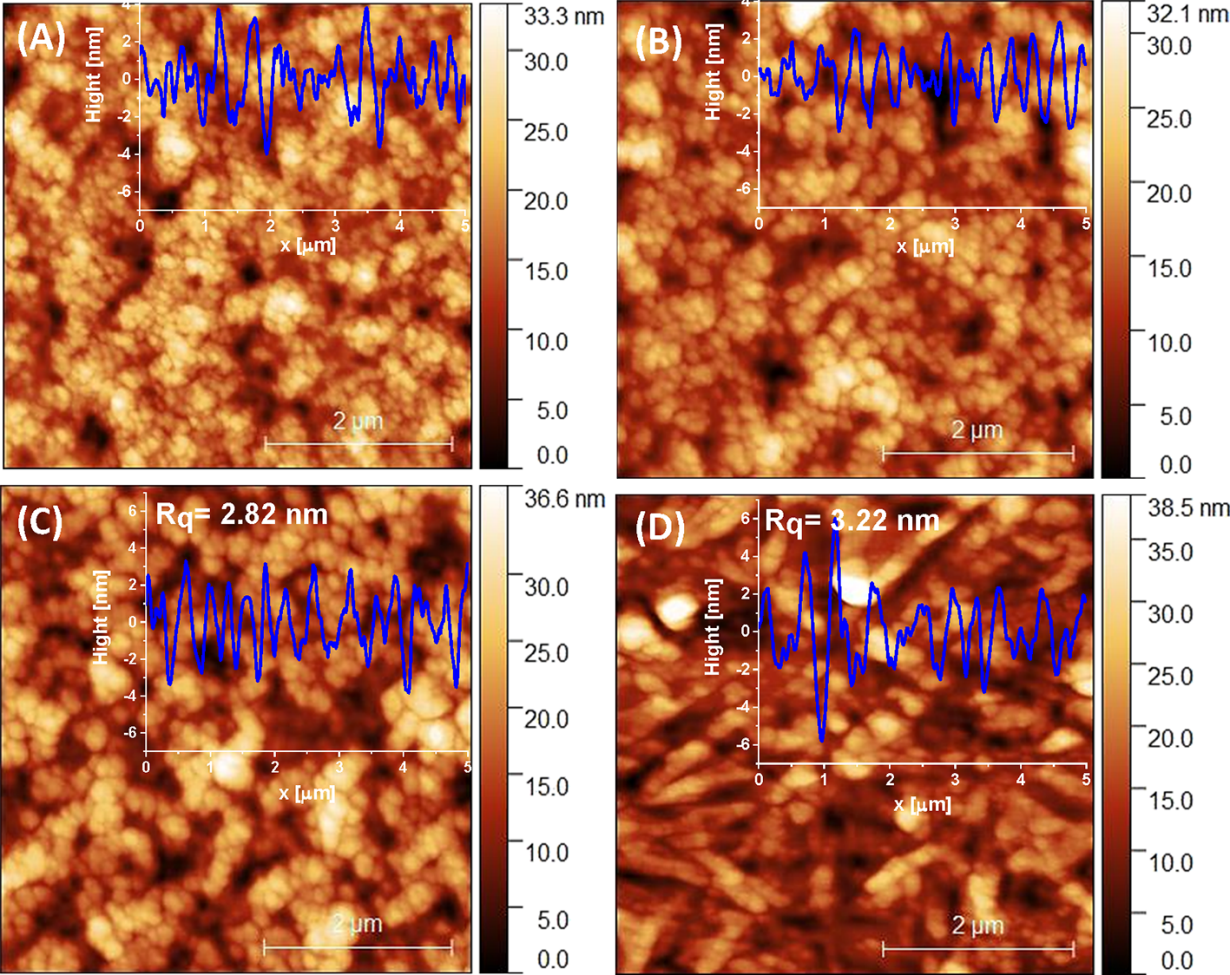
AFM images show the topography of TiO_2_ optical spacing layers deposited on top of P3HT:PCBM in a replica device configuration ITO/PEDOT/P3HT:PCBM with (A) no Ag NPs, (B) Ag NPs of 10 nm, (C) Ag NPs of 20 nm, and (D) Ag NPs of 30 nm. The inset shows a line scan of the surface topography used to calculate the film’s root-mean-square roughness (Rq), indicating the average surface topography of the film.

Figure 2 shows the 2D atomic force microscopy (AFM) images of TiO_2_ optical spacing films with and without Ag NPs of different diameters. The TiO_2_ film was deposited on top of the P3HT:PCBM photoactive organic blend using the layer configuration ITO/PEDOT/P3HT:PCBM, to simulate the conditions in the tested devices. The root-mean-square roughness (*R*_*q*_) was calculated by averaging the surface profile values of three-line scans (Figure S1, Supporting Information) using Gwyddion, a commercially available AFM analysis software. The surface topography of TiO_2_ shows variations in its surface roughness from 2 nm (with no Ag NPs) to 1.83, 2.82, and 3.22 nm with the addition of Ag NPs with diameters of 10, 20, and 30 nm, respectively.

**Figure 3:**
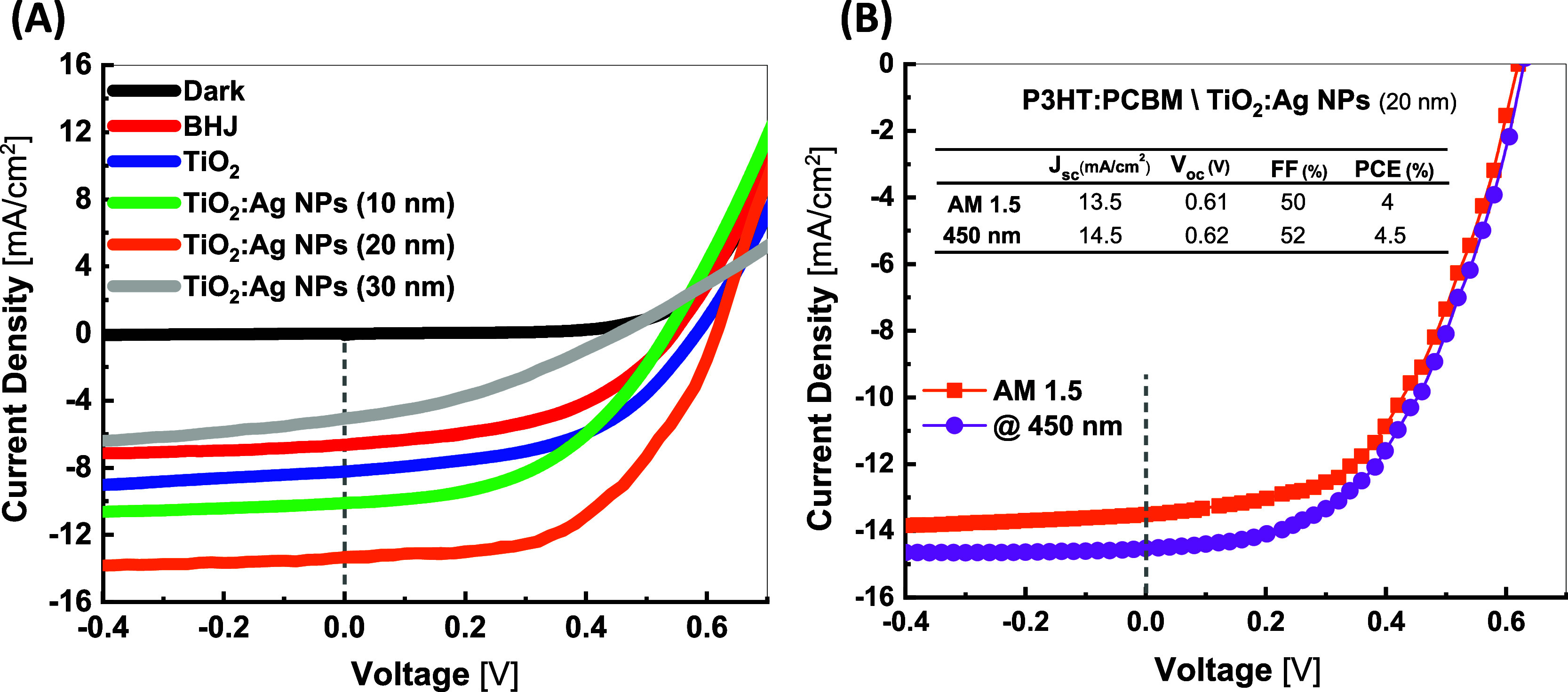
(A) *J*–*V* characteristics of BHJ solar cell devices with an evaporated Ag film as a back electrode and different plasmonic optical spacing layers (BHJ solar cell (no TiO_2_); TiO_2_; TiO_2_/ Ag NPs (10 nm); TiO_2_/ Ag NPs (20 nm) and TiO_2_/ Ag NPs (30 nm)) measured under AM1.5 illumination. (B) *J*–*V* comparison of BHJ solar cell incorporating the plasmonic optical spacer layer of TiO_2_/ Ag NPs (20 nm) measured under monochromatic light of 450 nm with the power of 55 mW.cm^–2^ (plasmonic resonance of Ag NPs embedded in TiO_2_).

The photovoltaic performance of the best five BHJ solar cells on illumination with the Air Mass 1.5 Global solar simulator (100 mW/cm^2^) is shown in Figure 3A. The BHJ device (red line) has no optical spacer film. The second device (blue line) incorporates a TiO_2_ film as an optical spacer. The performance of the remaining three devices (green, orange, and gray lines) prepared with Ag NPs of different diameters, 10, 20, and 30 nm, randomly embedded in separated TiO_2_ films. Table 1 provides a comparison of the devices’ photovoltaic performance at 1.5 AM conditions in terms of their open circuit voltage (*V*_*OC*_), short circuit current (*J*_*SC*_), fill factor (*FF*), and power conversion efficiency (*PCE*) values. The device with an optical spacing layer of TiO_2_ shows a *PCE* of 2.3%, a 53% enhancement compared to the device without an optical spacing layer, with efficiency of 1.5%. This improvement in device efficiency with the optical spacing layer has already been observed and attributed to the enhancement in the charge extraction process^57^ and the role of TiO_2_ in redistributing the electric field intensity inside the device, which helps to increase light absorption inside the active medium.^16,58^ Solar cells with a modified optical spacing layer of TiO_2_ with Ag NPs of 10 nm slightly enhance device efficiency to 2.4%. The highest efficiency of 4.0% is achieved with an optical spacer layer modified with 20 nm of Ag NPs with clear improvement in *J*_*SC*_ and *V*_*OC*_ of 13.5 mA/cm^2^ and 0.61 V, respectively. This is a 61% enhancement in *J*_*SC*_ and 6.3% in *FF* compared to device performance with only TiO_2_. The device performance is enhanced even further with *PCE* of 4.5% when using a monochromatic wavelength of 450 nm, matching the resonance absorption of Ag NPs, as shown in Figure 3B. A 6% and 2% enhancement in *J*_*SC*_ to 17 mA/cm^2^ and *FF* to 61% has also been reported under the illumination of 100 mW.cm^–2^ solar simulator when Au nanorods (30 nm length and 10 nm width) were incorporated between the Al back electrode and the TiO_*x*_ electron transporting layer in an organic solar cell based on PTB7:PCBM blend.^59^

**Table 1:** *J*–*V* Characteristics and Performance of Bulk Heterojunction Solar Cells with and without the Modified Optical Spacing Layers of Different Ag NPs Diameters Measured at AM1.5 Illumination Conditions

Device	*V*_OC_ [V]	*J*_SC_ [mA/cm^2^]	FF [%]	PCE [%]
BHJ	0.54	6.8	42	1.5 ± 0.18
TiO_2_	0.58	8.4	47	2.3 ± 0.13
TiO_2_:Ag_(10 nm)_	0.54	10	45	2.4 ± 0.16
TiO_2_:Ag_(20 nm)_	0.61	13.5	50	4.0 ± 0.13
TiO_2_:Ag_(30 nm)_	0.45	5.5	29	0.7 ± 0.11

Further increasing the size of Ag NPs to 30 nm, equivalent to the thickness of the optical spacing layer, led to a significant drop in *J*_*SC*_ and *V*_*OC*_ of 5.5 mA/cm^2^ and 0.45 V, respectively, and recording *PCE* of 0.7%. This deterioration in device performance with increasing the Ag NP size, reflected by the considerable reduction in the *FF*, has been observed before and attributed to the combinations of many factors, such as excitons recombination/trapping loss pathways aided by relatively large Ag NPs.^60−64^ The reduction in device efficiency could also result from Ag nanoparticles’ aggregation, which highly affects the surface roughness (as clearly illustrated in Figure 2D). Such aggregation could provide direct contact between the Ag NPs and the organic blend, creating charge recombination centers, which quench charge transport toward the cathode, causing a reduction in device efficiency.^65^ Furthermore, the high surface roughness, 3.22 nm with the addition of 30 nm Ag NPs, could invalidate the concept of an optical spacing layer in organic solar cells and negatively impact charge transportation and collection.^16,66^

**Figure 4:**
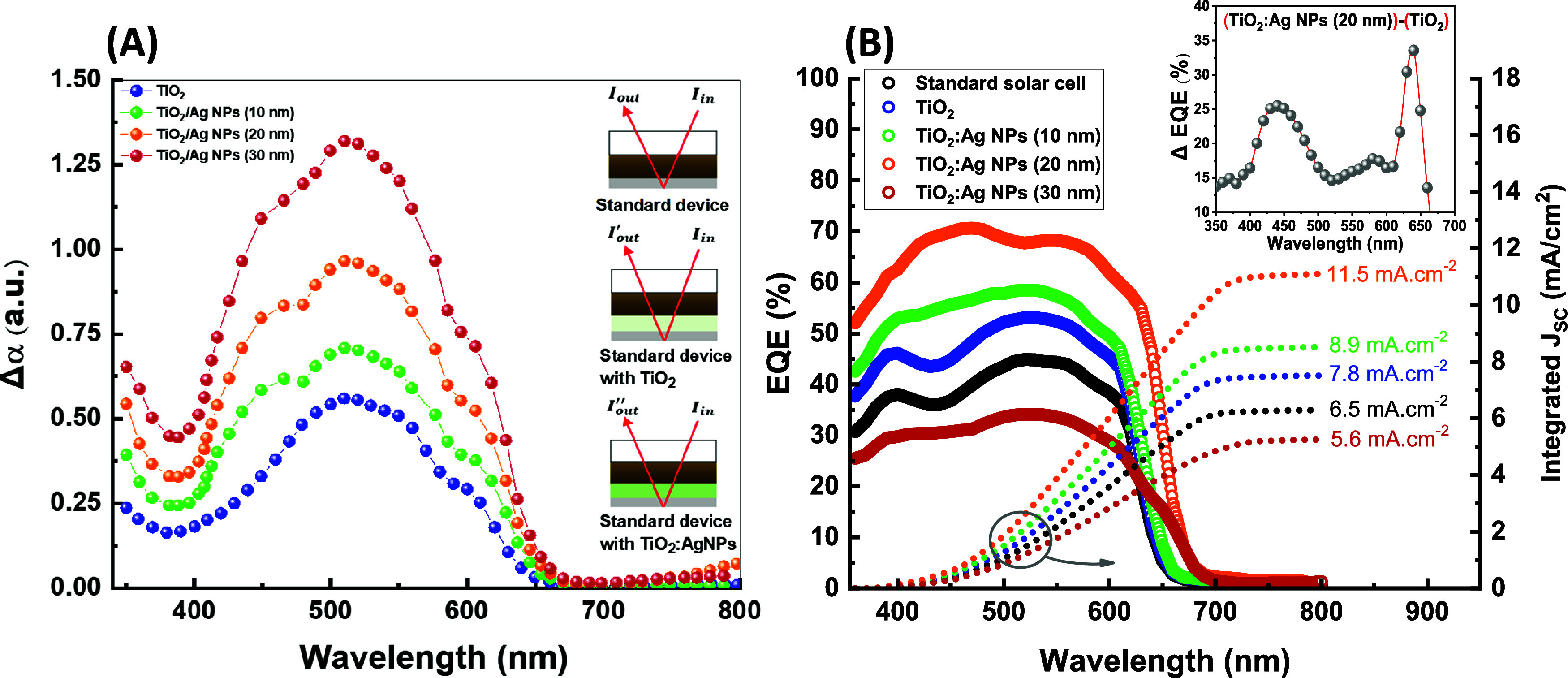
(A) Variation in the absorption spectrum (Δα) of devices with an optical spacing layer with respect to a device without a spacer. The inset shows a schematic illustration of the reflectivity measurements of the device structures (without the ITO and PEDOT: PSS), with *I*_in_ and *I*_out_ representing the intensity of the incident and reflected light, respectively. (B) External quantum efficiency (EQE) spectra of BHJ solar cells with and without the functional optical spacer layers and the *J*_SC_ integrated over the EQE. The inset shows the differential EQE calculated by subtracting the EQE of the device with the TiO_2_ layer from the TiO_2_:Ag NPs (20 nm) device.

To further investigate the role of the Ag NPs in the modified optical spacing layer on the device absorption efficiency, we measured the reflectivity of the BHJ solar cell with and without the optical spacing layer in a way similar to that reported by Lee et al.,^16^ with results shown in Figure 4A. The reflectivity spectra of glass/P3HT:PCBM/Ag, glass/P3HT:PCBM/TiO_2_/Ag, and glass/P3HT:PCBM/TiO_2_:Ag NPs/Ag were measured, and the difference in absorption is estimated according to the following relation:^16,58^
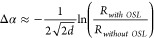
3where Δα is the variation in absorption of the glass/P3HT: PCBM/Ag device after introducing the modified OSL. *d* is the composite BHJ layer thickness (120 nm), *R*_*without OSL*_ and *R*_*with OSL*_ represent the reflection of the device structure without and with the optical spacer layer, respectively. The measured absorption spectrum gradually increases when modifying the optical spacing layer of TiO_2_ with 10, 20, and 30 nm Ag NPs.

To understand how the plasmonic effect contributes to the device performance, external quantum efficiency spectra (EQE) were collected from all OSC devices, as shown in Figure 4B. The BHJ device, without the optical spacing layer, shows a typical EQE response of the polymer: fullerene BHJ solar cell with a maximum of around 39% and 46% at 399 and 520 nm, consistent with the literature.^67−69^ With the insertion of the TiO_2_ optical spacer layer, the device shows an enhancement in its EQE, reaching about 54% over the entire exciting spectral region. After modifying the TiO_2_ layer with Ag NPs, the OSCs show further enhancement at the EQE, reaching 59% and 71% when adding Ag NPs with 10 and 20 nm diameters, respectively. Moreover, an enhancement of EQE is observed between the two peaks, developing into a distinct peak at about 460 nm for the 20 nm Ag NP sample. The inset shows the difference in integrated EQE enhancement after incorporating 20 nm Ag NPs into the optical spacing layer, which illustrates a distinct peak at around 450 nm that is directly correlated with the plasmonic resonance field of Ag NPs, as indicated earlier in the optical absorption, Figure 1A. Furthermore, a second broad peak is observed at longer wavelengths with a further enhancement at the edge of the solar cell’s absorption spectrum. These enhancements are investigated below by simulation.

The device with 30 nm Ag NPs shows a poor EQE performance of 35%, compared to the other devices, despite providing higher overall light absorption. This behavior is consistent with its substandard J-V characteristics shown in Figure 3A, a poor *J*_*SC*_ and *FF* of 5.5 mA/cm^2^ and 29 compared to 6.8 mA/cm^2^ and 42 of the pristine device. The EQE reflects the efficiency of charge generation, separation, and transportation within the device system, which is highly affected by the absorption response of the active layer. However, another decisive factor is the charge extraction process, which appears to be heavily disrupted by the incorporation of 30 nm Ag NPs. Relatively large Ag NPs have been found to adversely affect the electrical performance of the device due to undesired charge trapping/recombination centers and charge accumulation at the Ag NPs/active medium interface, which causes charge screening.^70,71^ It may also negatively interfere with the spatial internal field distribution aided by the optical spacing layer.^16^ The *J*_*SC*_ can also be calculated from the EQE integrated over all wavelengths (λ) using the following equation:
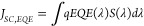
4Where *S*(λ) is the photon flux, and *q* is the charge of the carriers (Figure 4B), as reported by Saliba and Etgar.^72^ This value can deviate from those extracted from the J-V measurements at AM 1.5G due to the limited illumination bandwidth in the EQE measurements^73,74^ (Figure 4).

**Figure 5:**
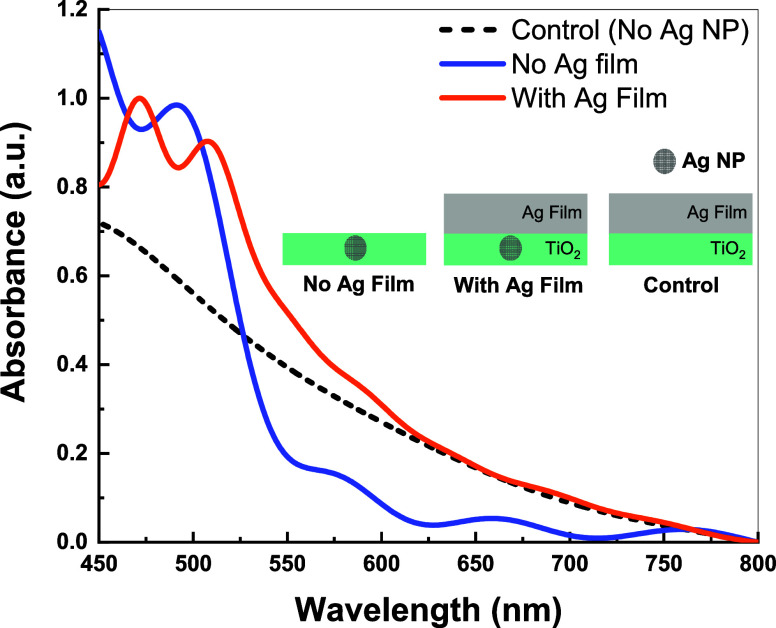
Calculated absorption of TiO_2_ incorporated 20 nm Ag NP with and without the Ag film and a TiO_2_/Ag film control structure. The inset depicts the simulated structures.

To further investigate the plasmonic contribution to the solar cell performance with 20 nm Ag NPs, FDTD simulations were performed to investigate the enhanced electric field stimulated by the plasmonic resonance absorption of an Ag NP embedded in TiO_2_. Figure 5 shows the calculated absorption of a TiO_2_:Ag NP with and without an overlying Ag thin film. A control simulation of TiO_2_/Ag film is also performed. With no Ag NP (control structure), the absorption spectrum shows a featureless line across the recorded spectral region with higher absorption at shorter wavelengths, attributed to the band absorption of TiO_2_. When the Ag NP is embedded in TiO_2_, the absorption spectrum shows an additional strong peak at 491 nm, attributed to the localized surface plasmon resonance. With an overlying Ag thin film, coupling between the localized plasmonic modes and the continuum of metal–semiconductor-metal gap modes results in two strong peaks at 476 and 521 nm, assigned to the plasmonic dipole mode and a higher-order multipole plasmonic mode, respectively.^45,75^Figures S2 and S3 (Supporting Information) show the calculated absorption spectra from simulations of a 20 nm Ag NP, placed at different vertical locations across a TiO_2_ thin film, with and without an overlying Ag film. They also show the corresponding spatial distribution of the plasmonic field enhancement for the different configurations. Comparing the two sets of spectra, it is evident that coupling to the overlying Ag film enhances the resonances in the nanogap between the NP and the Ag film and extends the spectra significantly toward the red. We believe such field coupling boosts light harnessing inside the BHJ solar device system and enhances performance. The split plasmonic resonance may explain why the incorporation of NPs into the OSL results in the observation of two rather than single broad peaks in the differential EQE spectra found in the inset of Figure 4B. The simulation was conducted at normal incidence, so TM coupled modes (E || z) were not excited. These modes, which are typically stronger and further red-shifted than the corresponding TE mode,^76^ can be accessed in the actual BHJ solar cell because of light scattering. They may be responsible for the further enhancement in EQE beyond 600 nm observed in the inset of Figure 4B. Indeed, a study of a BHJ solar cell incorporating a gold nanorod electron transport layer highlights the role of light scattering from the Au NPs penetrating inside the photoactive medium in the enhanced efficiency of the device.^59^

## Conclusions

We have significantly improved the efficiency of BHJ organic solar cells from 1.5% to 4.0% by incorporating Ag NPs in the TiO_2_ electron transporting and OSL of the device. The best performance was achieved when the Ag NP diameter, 20 nm, was two-thirds that of the thickness of the OSL. Differential EQE measurements show two broad spectral peaks that contribute to enhanced performance. FDTD simulations show that coupling the localized surface plasmonic resonance of the Ag NPs with the overlying Ag thin film cathode gives a similar two-peaked electric-field enhancement, extending the plasmonic effect over a broader spectral range. This increases the spectral overlap with the absorption spectrum of the BHJ layer and enhances the device’s performance. Our proposed structure provides a practical approach for plasmonic excitations within organic solar cell devices utilizing metallic NPs to enable an enhancement in power conversion efficiency. Additionally, the fabrication presented here, namely the solution process with self-assembly of nanoparticles, is easily scalable, making plasmonic enhancements in photovoltaics commercially viable.

## Supporting Information Available

2D AFM images of the surface profile of TiO_2_-incorporated Ag NPs of different sizes (10, 20, and 30 nm) were used to evaluate the surface roughness; FDTD simulations of the plasmonic field excitation of 20 nm Ag NP incorporated at different locations within the TiO_2_, with and without the Ag extended film (PDF)
