# Supplementary material for: Improving the Efficiency of Bulk-heterojunction Solar Cells through Plasmonic Enhancement within a Silver Nanoparticle-Loaded Optical Spacer Layer
Source: ACS Omega. 2025 Jan 13;10(3):2849–57. doi: 10.1021/acsomega.4c08801 (PMC11780413; doi:10.1021/acsomega.4c08801)
Supplement: Supplementary file 1 — ao4c08801_si_001.pdf [file ao4c08801_si_001.pdf]

Supporting Information

Improving the efficiency of bulk-heterojunction solar cells through plasmonic enhancement within a silver nanoparticle-loaded optical spacer layer

Mohammed A. Ibrahem*[1], Bassam G. Rasheed[2], Betul Canimkurbey[3,4], Ali M. Adawi*[5], Jean-Sebastien G. Bouillard*[5], and Mary O'Neill*[6]

[1] Laser Sciences and Technology Branch, Applied Sciences Department, University of Technology, Baghdad 10066, Iraq.

[2] Laser and Optoelectronics Engineering Department, College of Engineering, Al Nahrain University, Baghdad, Iraq.

[3] UNAM − Institute of Materials Science and Nanotechnology and National Nanotechnology Research Center, Bilkent University, Ankara 06800, Turkey.

[4] Serefeddin Health Services Vocational School, Central Research Laboratory, Amasya University, Amasya 05100, Turkey.

[5] Department of Physics and Mathematics, University of Hull, Kingston upon Hull HU67RX, United Kingdom.

[6] School of Science and Technology, Nottingham Trent University, Clifton Lane, Nottingham NG11 8NS, United Kingdom.

mohammed.a.ibrahem@uotechnology.edu.iq, a.adawi@hull.ac.uk, j.bouillard@hull.ac.uk, and mary.oneill@ntu.ac.uk

Keywords: *Bulk heterojunction solar cells, plasmonic-nanogap, silver nanoparticles, TiO_2 optical spacer layer*

Simulation work

Finite-difference time-domain (FDTD) simulations provided by Lumerical Solutions software were used to study the optical absorption enhancement in the solar cell device and the optical spacing layer of TiO_2 with a thickness of 30 nm after incorporating 20 nm Ag NPs. We monitored the resonant near-field enhancement of a single 20 nm Ag NP embedded in the TiO_2 electron transporting layer at different locations from the 100 nm Ag back electrode film. The simulations used a refined uniform mesh around the nanoparticle and the interaction region. The incident light, with a bandwidth of 450 to 800 nm having a plane wave, propagates the structure from the y-direction (normal to the plane of the thin films matching the experimental configuration) with the electric field polarization recorded in the x-direction. Periodic boundary conditions were used for the lateral dimensions of the proposed simulation structures. Data from the CRC Handbook of Chemistry and Physics described the complex refractive indices of silver nanoparticles and TiO_2. A total field scattered field (TFSF) monitor configuration was used to record the absorption spectra of Ag NP within the proposed structure.

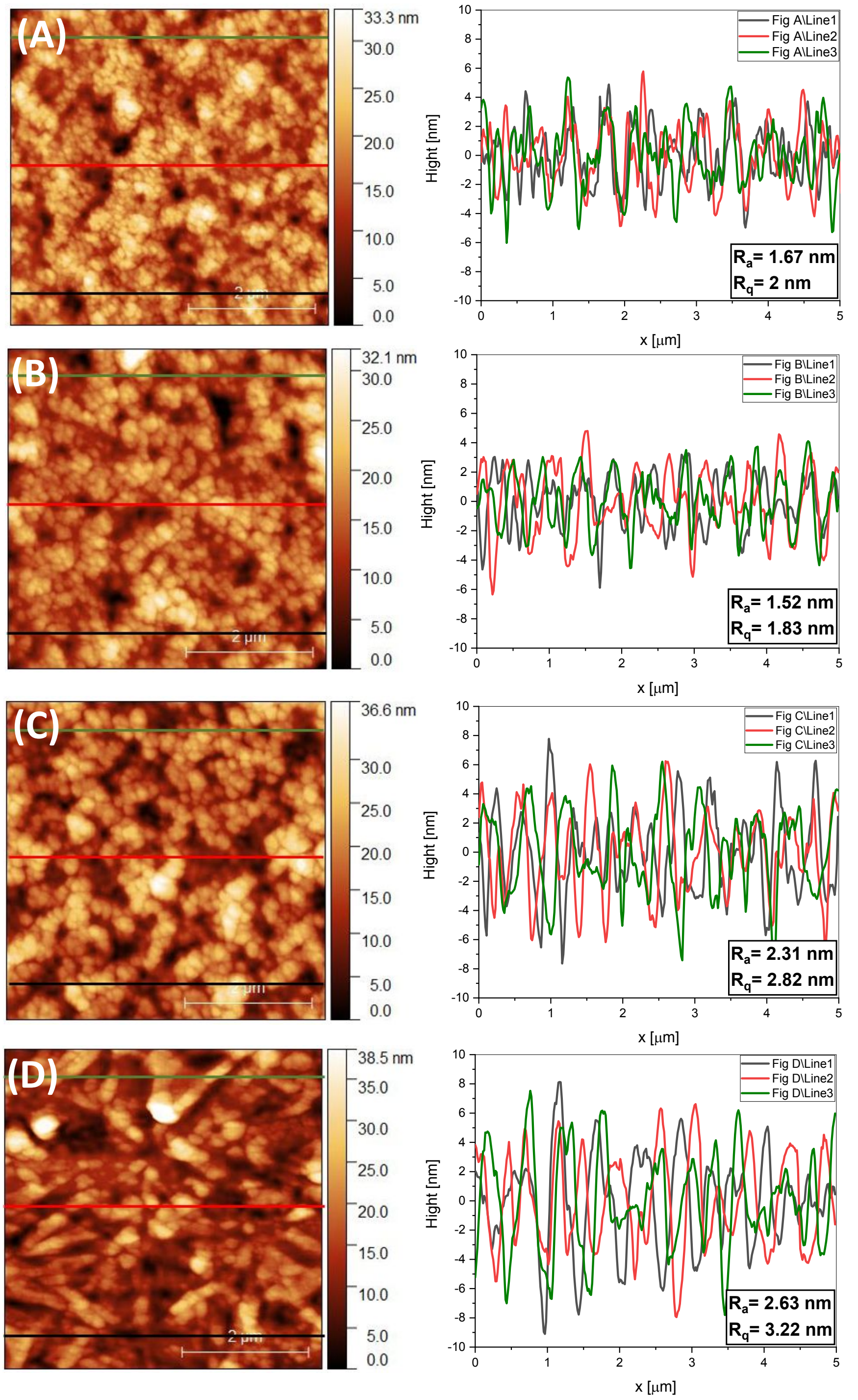

Figure S1: AFM images in 2D and 3D as well as their surface profile scans of TiO_2 films (A) without Ag NPs, and with (B) 10 nm Ag NPs, (C) 20 nm and (D) 30 nm. Surface roughness values were averaged for the three-line scans.

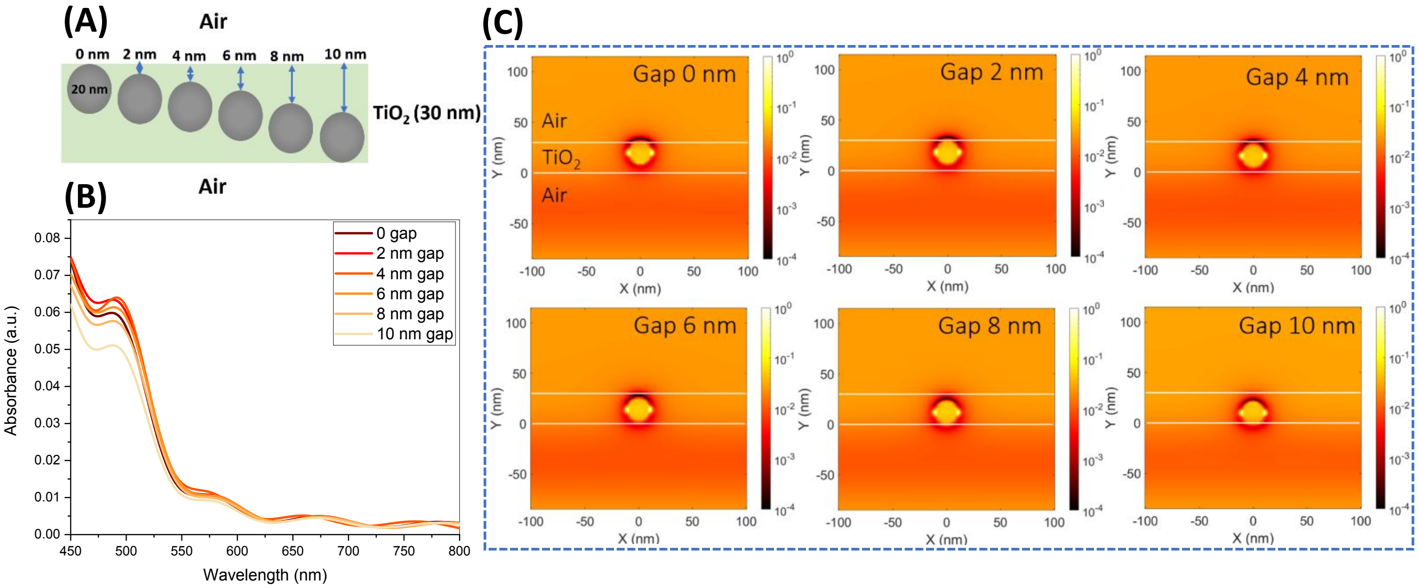

Figure S2: (A) Schematic illustration of the simulated structure showing the Ag NPs with a diameter of 20 nm located at different locations within the 30 nm optical spacing layer of TiO_2. (B) The calculated absorption of the Ag NP illustrated in (A). (C) The field distribution of the Ag NP at different locations within the optical spacing layer of TiO_2, illuminated from the bottom side, at the excitation wavelength of 488 nm.

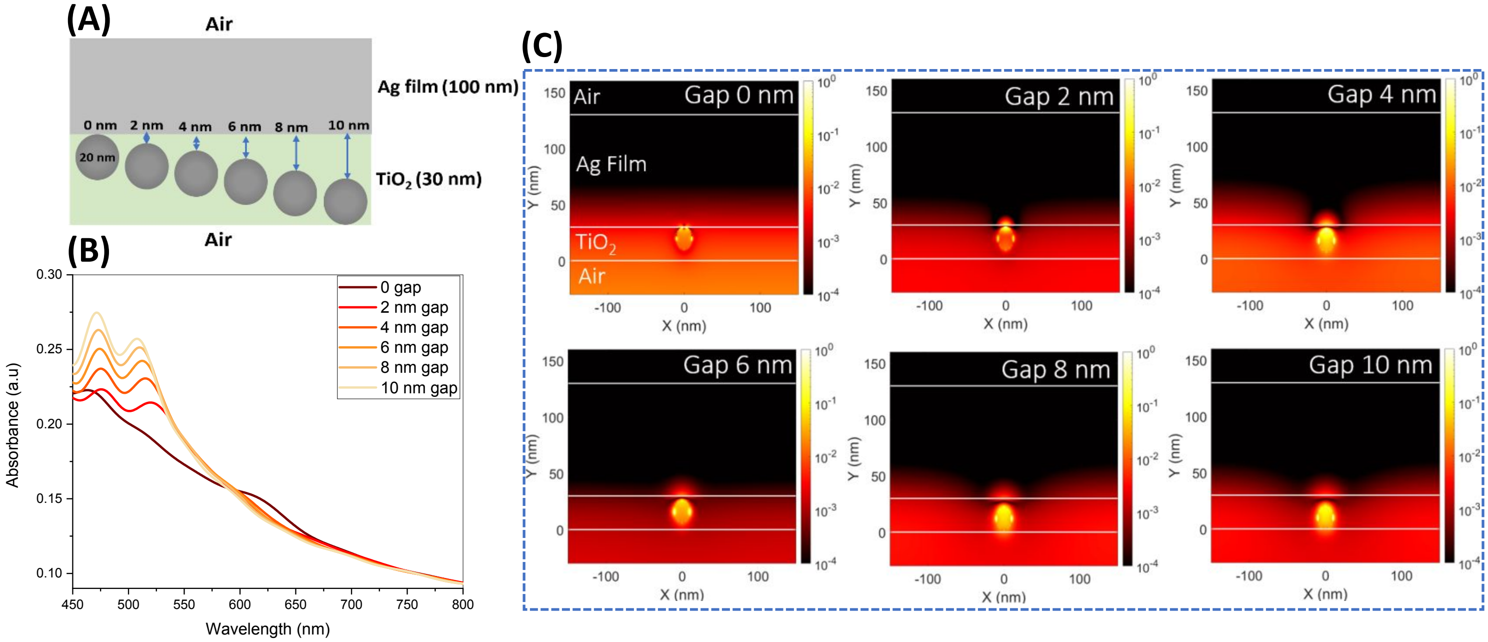

Figure S3: (A) Schematic illustration of the simulated structure showing the Ag NPs with a diameter of 20 nm, located at different locations from the Ag extended film within the 30 nm optical spacing layer of TiO_2. (B) The calculated absorption of the Ag NP illustrated in (A). (C) The field distribution of the Ag NP at different locations away from the Ag film within the optical spacing layer of TiO_2 at the excitation wavelength of 476 nm illuminated from the bottom side.
